# Ferroptosis: Regulated Cell Death

**DOI:** 10.2478/aiht-2020-71-3366

**Published:** 2020-06-29

**Authors:** Ivana Čepelak, Slavica Dodig, Daniela Čepelak Dodig

**Affiliations:** 1 University of Zagreb, Faculty of Pharmacy and Biochemistry, Department of Medical Biochemistry and Haematology, Zagreb, Croatia; 2 Croatian Institute of Public Health, Department of Toxicology, Zagreb, Croatia

**Keywords:** glutathione peroxidase 4, iron, lipid peroxidation, reactive oxygen species, system Xc^−^, glutation peroksidaza 4, lipidna peroksidacija, reaktivne kisikove vrste, sustav Xc-, željezo

## Abstract

Ferroptosis is a recently identified form of regulated cell death that differs from other known forms of cell death morphologically, biochemically, and genetically. The main properties of ferroptosis are free redox-active iron and consequent iron-dependent peroxidation of polyunsaturated fatty acids in cell membrane phospholipids, which results in the accumulation of lipid-based reactive oxygen species due to loss of glutathione peroxidase 4 activity. Ferroptosis has increasingly been associated with neurodegenerative diseases, carcinogenesis, stroke, intracerebral haemorrhage, traumatic brain injury, and ischemia-reperfusion injury. It has also shown a significant therapeutic potential in the treatment of cancer and other diseases. This review summarises current knowledge about and the mechanisms that regulate ferroptosis.

## Abstract (HR)

Feroptoza je nedavno identificirani oblik regulirane stanične smrti koji se od ostalih poznatih oblika stanične smrti razlikuje morfološki, biokemijski i genetski. Glavna svojstva feroptoze uključuju slobodno redoks aktivno željezo i posljedičnu, o željezu ovisnu, peroksidaciju polinezasićenih masnih kiselina u fosfolipidima staničnih membrana te gubitak aktivnosti glutation peroksidaze 4, što rezultira akumulacijom lipidnih, reaktivnih kisikovih spojeva. Feroptoza se sve više povezuje s raznim bolestima kao što su neurodegenerativne bolesti, karcinom, moždani udar, intracerebralna krvarenja, traumatične ozljede mozga i ishemijsko-reperfuzijska ozljeda. Također je pokazan značajan terapijski potencijal u liječenju raka i drugih bolesti. Ovaj pregled sažima trenutačne spoznaje i mehanizme koji reguliraju feroptozu.

According to the Nomenclature Committee on Cell Death (NCCD), there are two main types of cell death, depending on molecular events or biochemical mechanisms that have led to it (1): a) accidental cell death (ACD) and b) regulated cell death (RCD). The first type is the consequence of unexpected chemical, mechanical, and physical stress (e.g. heat-shock, freeze-thawing) that overcomes the existing control mechanisms. It is characterised by distinct morphological features, impaired membrane permeability, organelle dilatation, dissociation of ribosomes, and release of damage-associated molecular patterns (DAMPs) and is insensitive to pharmacological or genetic interventions (death by so-called “sabotage” programme). The RCD type is regulated by precise molecular mechanisms (death by so-called “suicide programme”) (2, 3). NCCD also defines the term programmed cell death (PCD) as a subset of RCD. This is a physiological form of cell death at the genetic and biochemical level first proposed in 1972 (4, 5) and is essential for the development of living organisms and maintenance of homeostasis.

RCD is often synonymous with caspase-dependent apoptosis (apoptotic cell death), but there are many non-apoptotic forms of RCD such as necroptosis, pyroptosis, ferroptosis, parthanatos, autophagy-dependent cell death, alkaliptosis, and oxytosis (6). These differ from one another in biochemical, functional, and morphological terms (7). Table 1 lists major RCD forms, but there are others, such as cellular senescence (irreversible inhibition of the cell cycle) (1, 12), alkaliptosis (cell death triggered by intracellular alkalinisation) (6), lysosome-dependent cell death (mediated by hydrolytic enzymes released into the cytosol after lysosomal membrane permeabilisation) (6, 13), entotic cell death (a form of cell cannibalism in which one cell devours and kills another and occurs mostly in epithelial tumour cells) (14), and netotic cell death [mediated by the release of neutrophil extracellular traps (NETs) – extracellular net-like DNA-protein structures released by cells in response to infection or injury] (15).

**Table 1:** Some forms of regulated cell death and their properties

Cell type death	Definition	Morphological (and biochemical) features	References
Apoptosis	Apoptosis is a prevailing form of RCD that requires activation of caspases leading to DNA fragmentation without loss of plasma membrane integrity. It can be extrinsic and intrinsic.	Membrane blebbing, cell shrinkage, retraction pseudopods, reduction of cellular and nuclear volume, nuclear fragmentation, chromatin condensation, apoptotic body formation (activation of caspases, e.g. CASP2, CASP8, CASP3, oligonucleosomal DNA fragmentation, cytochrome c release altered Bc1-2 family protein expression and activation)	6, 8, 9
Ferroptosis	Ferroptosis is a form of cell death caused by iron-dependent lipid peroxidation and L-ROS accumulation.	Normal spherical cells-lack of rupture and blebbing of the plasma membrane, rounding up of the cell, small mitochondria with condensed mitochondrial membrane densities, reduction or vanishing of mitochondria cristae, as well as outer mitochondrial membrane rupture, normal nuclear size (L-ROS accumulation, activation of MAPKs, inhibition of system Xc- with decreased cystine uptake, GSH depletion and increased NADPH oxidation, inhibition GPX4, release of AA mediators (e.g. 11-HETE and 15-HETE).	8, 9, 10
Pyroptosis	Pyroptosis is a form of lytic cell death that occurs in inflammatory cells in response to proinflammatory stimuli.	Inflammasome activation membrane rupture, cell swelling/cell oedema and lysis, pore-induced intracellular traps, DNA fragmentation, nuclear condensation (DAMP release-e.g.HMGB1, ATP), dependent on caspase 1 and 7, proinflammatory cytokine release.	9, 10
Necroptosis	Necroptosis is a programmed form of necrosis, a modality of RCD that is activated by RIPK1 and requires RIPK3-dependent phosphorilation of MLKL.	Plasma membrane rupture, cell swelling and lysis, organelle swelling, moderate chromatin condensation (ATP decline, DAMPs release, activation of RIPK1, RIPK3 and MLKL, PARP1 hyperactivation).	6, 8, 10, 11
Parthanatos	Parthanatos is a PARP1-dependent RCD that is activated by oxidative stress-induced DNA damage and chromatinolysis.	Cell swelling and lysis, DNA fragmentation, nuclear condensation, release of mitochondrial apoptosis-inducing factor [excessive activation of PARP1, caspase-independent NAD+ and ATP depletion, accumulation of poly ADPribose (PAR) polymers, AIFM1 release from mitochondria to nucleus].	6,9
Autophagy- dependent cell death	Autophagy-dependent cell death is a type of RCD dependent on autophagy machinery and/or components of autophagy.	Autophagic vacuolisation, lack of cell membrane changes, lack of chromatin condensation, accumulation of double-membraned autophagic vacuoles, increased lysosomal activity, MAP1LC3B-I, MAP1LC3-II (or LC3B-I, LC3B-II) conversion, substrate degradation (e.g. p62).	6, 8, 9, 10

AA – arachidonic acid; AIF – apoptosis-inducing factor; AIFM1 – apoptosis inducing factor mitochondria-1; ATP –adenosine triphosphate; CASP – caspase; DAMP – damage-associated molecular patterns; DNA – deoxyribonucleic acid; GPX4 – glutathione peroxidase 4; GSH – glutathione reduced form; 11-HETE – 11-hydroxyeicosatetraenoic; HMBG1 – high-mobility group box 1 ; MAP1LC3B – microtubule-associated protein 1 light chain 3 beta; MAPK – mitogen-activated protein kinase; NAD – nicotinamide adenine dinucleotide; NADPH – nicotinamide adenine dinucleotide phosphate; PARP1 – poly(ADP-ribose) polymerase 1; RCD – regulated cell death; RIPK1 – receptor interacting serine/threonine protein kinase 1; ROS – reactive oxygen species

Cell death can generally result in immune response to dead cell antigens, commonly known as “immunogenic cell death” (16). An important role is attributed to the release of DAMPs, which include high-mobility group box 1 (HMGB1) proteins, histones, mitochondrial transcription factor A (TFAM), and non-proteaceous entities such as DNA, RNA, and extracellular ATP (17) from dead or dying cells. Cellular programmes associated with the immune component are apoptosis, necroptosis, ferroptosis, pyroptosis, and parthanatos (9).

Necrosis, as a form of ACD, is characterised by a rupture of the plasma membrane, cell swelling (oncosis), a decrease in energy and release of DAMP, which leads to cell lysis and consequent propagation of inflammation (6, 9). A variant of necrosis has also been described to involve mitochondrial permeability transition, pore opening characterised by plasma membrane rupture, cell swelling and lysis, energy decline, DAMP release, and mitochondrial swelling (9).

The nature, regulation, and physiological and pathological relevance of various cell death programmes continues to be at the centre of research interest. For the last few decades, scientific interest has particularly been focused on the features and molecular mechanisms of ferroptosis, which seems to be involved in both health and disease states (3). The aim of this review is to present the latest insights into this form of cell death, including its main mechanisms of action and possibilities of manipulation.

The data selected for this review were collected by searching the PubMed database for articles published in English between 2000 and 2020 using the following terms: cell death, regulated cell death, ferroptosis, lipid peroxides, iron, and glutathione peroxidase. In addition, we ran a search for possible mechanisms and physiological and pathophysiological significances using the following specific terms: programmed cell death, labile iron pool, apoptosis, and erastin.

## The concept

As a new form of cell death, ferroptosis (from Latin *ferrum* for iron and Greek *ptosis* for decline/failure) was first described in 2012 (18), although some characteristic changes had been known earlier (19). It is an iron-dependent form of RCD or non-apoptotic, caspase-independent cell death with necrotic morphology caused by lipid reactive oxygen species (L-ROS; or lipid peroxides) accumulated in cell membranes through iron-mediated lipid peroxidation. It is an adaptive form of RCD, which means that it depends on metabolic conditions in the cell (20). It is also considered a pro-inflammatory, immunogenic form of RCD, since DAMPs are released during this process (21). According to its genetic, morphological, and biochemical characteristics, this process significantly differs from other forms of RCD. For example, ferroptotic cells have smaller mitochondria, higher mitochondrial membrane density, negligible mitochondrial crystals, and mitochondrial membrane rupture (Table 1). Some studies suggest that ferroptosis still shares several biochemical features with oxytosis (22), necroptosis (23), and autophagy or ferritinophagy (24, 25). This indicates some interdependence between these cell death programmes, but further research is needed in this regard.

**Figure 1:**
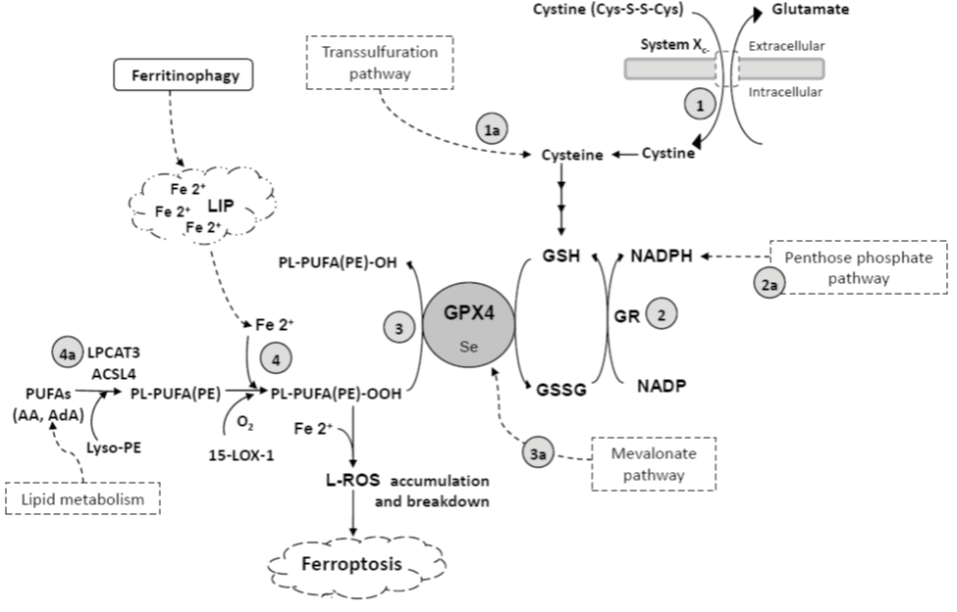
Proposed cellular mechanisms of ferroptosis. Ferroptosis occurs when the reduction of PL-PUFA (PE)-OOH *via* GPX4 is insufficient to prevent iron-mediated L-ROS accumulation. An important cysteine supply route for GSH synthesis is the import of cystine via the antiporter system Xc^-^ (1). An additional or alternative cysteine route is transsulphuration (1a). GSSG is reversed into GSH with the catalytic action of NADPH-dependent GR (2) generated in the penthose phosphate patway (2a). GSH is a cofactor of the GPX4 enzyme that prevents the accumulation of PL-PUFA(PE)-OOH by converting them to PL-PUFA(PE)-OH (3), and in the process of GPX4 maturation participates IPP from the mevalonate pathway (3a). Free redox active iron is involved in the formation of L-ROS from membrane PL-PUFA(PE) through the Fenton reaction and oxidation by15-LOX-1 (4). ACSL4 and LPCAT3 enzymes are involved in the formation of PL-PUFA (PE) (4a). AA – arachidonic acid; ACSL4 – acyl-CoA synthetase long chain family member 4; AdA – adrenic acid; GPX4 – glutathione peroxidase; GR – glutathione reductase; GSH – glutathione reduced form; GSSG – glutathione disulfide; IPP – isopentenyl pyrophosphate; LIP – labile iron pool; 15-LOX-1 – 15-lipooxygenase-1; LPCAT3-- lysophosphatidylcholine acyltransferase; L-ROS – lipid reactive oxygen species;PE – phosphatidylethanolamine; NADP – nicotinamide adenine dinucleotide phosphate; NADPH – reduced nicotinamide adenine dinucleotide phosphate; PL-PUFA(PE)-OH – lipid alcohol; PL-PUFA(PE)-OOH – PUFA-containing phospholipid hydroperoxides; PL-PUFA(PE) – PUFA-containing phospholipids; PUFA – polyunsaturated fatty acid; Se – selenocysteine

The mechanism of ferroptosis that was discovered the first by *in vitro* studies (18, 20) was the one with erastin, a small molecule that inhibited the cystine/glutamate antiporter system Xc^-^. This depleted cysteine required for the synthesis of antioxidant glutathione (GSH), a cofactor of the glutathione peroxidase-4 enzyme (GPX4; also known as phospholipid hydroperoxide glutathione peroxidase, PHGPx), which protects cells from L-ROS accumulation by reducing polyunsaturated fatty acids containing phospholipid hydroperoxides (PL-PUFA(PE)-OOHs (or lipid-hydroperoxides, L-OOHs) to the corresponding lipid alcohols and by limiting further formation of highly reactive alkoxyl radicals (L-OO*). The result of reduced GPX4 activity is the accumulation of toxic levels of PL-PUFA(PE)-OOH within the cell (18, 20). Figure 1 summarises the main pathways of ferroptosis.

According to literature, lipid peroxidation, which is a key factor in ferroptosis, involves various cell organelles, plasma membranes, endoplasmic reticulum, lysosomes, and mitochondrial membranes (26, 27), but opinions differ (18, 28). The susceptibility of individual cell organelles to lipid peroxidation is generally considered to depend on the “pool” of lipids in each organelle, iron storage, GSH level, and lipoxygenase (LOX) expression, which differs between cell types. Lipid peroxidation requires certain polyunsaturated fatty acids (PUFAs) in phospholipids (PL), whose production is related to iron metabolism and other factors. In other words, cell susceptibility to ferroptosis correlates with the abundance of PL acetylated with PUFA-PLs that can easily oxidise, the presence of free, redox-active iron, and inefficient removal of PL-PUFA(PE)-OOH (defective GSH-GPX4 system). In broader terms, ferroptosis is associated with iron, lipid metabolism, and some amino acids (18, 29, 30), which explains relatively large differences in susceptibility to ferroptosis between different cell types. Susceptibility to ferroptosis could therefore be altered by the expression and activity of proteins and pathways controlling the levels, transport, storage, and metabolism of iron, PUFAs, cystine, cysteine, GSH, glutamine, and selenocysteine (20).

## Major pathways regulating ferroptosis

Iron-dependent lipid peroxidation is considered to be the key to ferroptosis (31). However, this process can also be triggered by physiological conditions such as high extracellular glutamate, small molecules that block cystine import into the cell by the antiporter system Xc^-^, molecules that initiate degradation or covalently inhibit GPX4, and genetic deletion of GPX4 (18, 32, 33).

The following is a concise description of the major pathways of ferroptosis: GPX4 inactivation, L-ROS accumulation, and presence of redox active iron.

### Inactivation of GPX 4

GPX4 activity may be lowered by direct enzyme inhibition (loss of activity or stimulation of enzyme protein degradation) or inhibition of the antiporter system Xc^-.^ In the first case, inhibition is most often mediated by RAS-synthetic lethal 3 (RSL3), an alkylating molecule that irreversibly binds to selenocysteine in the active site of GPX4 (30), but there are other inducers of ferroptosis that can deplete or degrade the enzyme protein, such as ferroptosis inducer 56 (FIN56) and caspase independent lethal 56 (CIL56), a molecule that causes non-apoptotic cell death through an acetyl-CoA-carboxylase-1-dependent process (20, 34). Ferroptotic cell death can also be induced through genetic inhibition of GPX4 by siRNA (35).

As for the other pathway – the inhibition of system Xc^-^ – here is how this membrane-based, sodium-independent and chloride-dependent cystine/glutamate antiporter system works: it imports extracellular cystine in exchange for intracellular glutamate (36) and through the catalytic action of cystine reductase transforms cystine into cysteine, which is required for the synthesis of GSH and subsequently GPX4. Under normal conditions GPX4 catalyses the reduction of PL-PUFA(PE)-OOHs into alcohols (37). When this system is inhibited by small molecules such as erastin and its analogues (piperazine erastin and imidazole ketone erastin) and/or sorafenib, GSH levels drop, GPX4 is inactivated, and L-ROS starts to accumulate (18, 30).

### Accumulation of L-ROS

Aerobic organisms are continuously exposed to various reactive oxygen species (ROS) such as superoxide radicals (*O_2_^-^), hydrogen peroxide (H_2_O_2_), hydroxyl radical (*OH), and lipid peroxides (L-ROS) such as L-OOH, peroxyl (L-OO*), and L-O* radicals (38). While low, controlled L-ROS levels are acceptable for normal cell and organism functions, higher levels are associated with numerous chronic degenerative processes and acute organ injuries. These conditions are the result of an oxidant-antioxidant imbalance that results in oxidative stress.

The formation of cellular L-ROS involves iron-catalysed spontaneous chain reaction generating toxic radicals (non-enzymatic process) (20, 39) and enzyme-mediated oxidation of PUFAs (40, 41). Lipid compounds that are the most sensitive to lipid peroxidation and are thus involved in triggering ferroptosis are PL-PUFAs, arachidonic acid (AA) in particular, and the elongation product of adrenic acid (AdA) (29).

The formation of PL-PUFAs is mediated by the acyl-CoA long chain synthetase 4 (ACSL4), which catalyses the formation of acyl CoA derivatives (AA-CoA and AdA-CoA). Another enzyme, lysophosphatidylcholine acyltransferase 3 (LPCAT3), esterifies these derivatives into phosphatidylethanolamine (PE) forms AA-PE and AdA-PE that are inserted into the PL membrane. The resulting PL-PUFA(PE) produces L-ROS, which in turn executes ferroptosis (7).

PL-PUFA(PE) oxidation takes place in stages. In the first stage, *OH radicals attack PUFA on the bis-allylic position to create carbon-centred radicals that can react with molecular oxygen and produce L-OO*. In the second stage, L-OO* propagates by abstracting hydrogen from another PL molecule, which leads to the formation of PL-PUFA(PE)-OOH. L-OO* may also be added to the bis-allylic position of another PL molecule and produce a PL-OO-PL dimer. In the third stage, the reaction is continued until two radicals come together and form a non-radical molecule or the chain reaction is interrupted by some lipophilic antioxidant. If weakly bound or if free iron (Fe^2+^, Fe^3+^) is present (42, 43), PL-PUFA(PE)-OOHs can undergo reductive cleavage producing a toxic lipid LO* radical. These lipid radicals can abstract protons from neighbouring PUFAs and start a new cycle of lipid oxidation and damage (44). In addition, secondary lipid peroxidation products can be generated by intramolecular rearrangement and cleavage of PL-PUFA(PE)-OOH such as malondialdehyde (MDA), 4-hydroxynonenal (4HNE), and oxygenated PLs (33).

Enzyme-mediated formation of PL-PUFA(PE)-OOH (35) involves lipid-peroxidising enzymes that contain mononuclear iron centres and can easily receive iron from poly-rC binding chaperone proteins (PCBPs). Their major substrates are AA and linoleic acid. The enzymes are classified according to their positional specificity for AA oxygenation to 5-, 8-, 12-, 15-LOX. They catalyse the introduction of molecular oxygen into PUFAs to produce metabolites of 15-hydroxyeicosatetraenoic acid and 13-hydroxyoctadecadienoic acid (45, 46). The contribution of enzymatically and non-enzymatically mediated lipid peroxidation to ferroptosis differs (41, 47).

If the resulting L-ROS forms are not successfully detoxified by GPX4, they accumulate in cell and organelle membranes, which results in ferroptosis and membrane disruption. Although the exact mechanism is not clear, it is assumed that “hydrophilic pores” formed on membranes change membrane permeability and lay grounds for the “osmotic catastrophe” (48). Another assumption is that the resulting secondary, lipophilic electrophiles (e.g. MDA) may act as downstream signalling molecules and undetected protein effectors (49, 50).

### Presence of redox-active iron

Current knowledge indicates that LOX enzymes and the non-enzymatic Fenton reaction contribute the most to lipid peroxidation and L-ROS accumulation in ferroptosis (51, 52). The Fenton reaction involves oxidation of the ferrous to ferric form of iron and electron transfer to H_2_O_2_ (Fe^2+^+H_2_O_2_**→**Fe^3+^+*OH+HO^-^) producing a very reactive *OH radical (ferric iron from the reaction can be reduced to ferrous iron in the presence of O*_2_^-^ in the Haber-Weiss reaction).

Iron is an essential transition metal for all life forms on Earth. It is necessary for erythrocytopoiesis, for numerous enzymes involved in DNA replication, translation, and repair, for antimicrobial oxidative burst, and for many other biological processes, most often in the form of Fe-S-clusters (53). Thanks to its property to reversibly lose or receive electrons and transit from one valence state to another, it catalyses various biochemical reactions. The presence of free intracellular iron can therefore strongly influence cellular redox status and contribute to oxidative stress in cells. At the cellular level, iron homeostasis is regulated by a post-transcriptional mechanism mediated by the iron responsive protein/iron responsive element (IRP-IRE) system, while at the systemic level (absorption, utilisation, storage, and recycling) it is regulated by the hepcidin-ferroportin feedback loop and numerous other proteins such as divalent metal transporter (DMT1), ferrireductase, haem-oxygenase-1 (HMOX-1), ferroportin, transferrin (Tf), transferrin receptor-1 (TfR1), mitoferrin 1, and frataxin (54, 55, 56).

Under normal conditions, iron is delivered to the cell by Tf and TfR1.There ferric iron is reduced to its ferrous form, which is then introduced into the cytosol by DMT1. If there is excess free, non-transferrin-bound iron (NTBI), it is imported by transmembrane transporter proteins ZIP8 and ZIP 14 (57). Within the cells, iron binds to multifunctional PCBPs, which prevent iron from becoming part of the redox-active intracellular “labile iron pool” (LIP) and eventually its cytotoxic effects (57). In erythroid cells, most of the iron from LIP is transported into mitochondria by mitoferrin 1 and 2 and is stored as mitoferritin or incorporated into haem and Fe-S clusters, required for all electron transport chain complexes (57). In non-erythroid cells, iron from the LIP or the one bound to PCBPs is stored in the form of ferritin. When the stored iron is mobilised, ferritin binds to the autophagic nuclear receptor coactivator 4 (NCOA4). NCOA4 transports it to lysosomes, where it is degraded and released in a process called ferritinophagy (25). Ferritinophagy plays a major role in the recycling of intracellular redox-active iron, which also emphasizes the importance of lysosomes for ferroptosis (25). The resulting radicals and lipid peroxidation end products are highly reactive and cause massive oxidative damage, which is not exactly consistent with the regulated nature of ferroptosis.

Iron-dependent oxidative metabolism has been an indispensable part of life for billions of years. In view of this well-known fact, it was interesting to see that one research from 2010 (57), that is, before ferroptosis was recognised as a process, associated it with cell death. Degenerative changes in many diseases have been found to coincide with dysregulation of iron metabolism (57). Further research will show how consistent these findings are with the demonstrated mechanisms of cell death by ferroptosis *in vivo* and whether ferroptosis is the oldest and in many contexts the most important form of RCD.

For now, it can be concluded that lipid peroxidation mediated by free redox active iron plays a central role in ferroptosis (18). However, the precise role of iron in this process is yet to be determined. What we know is that iron catalyses the formation of L-ROS through the Fenton reaction and/or by iron-dependent LOX (58). The second assumption involves iron-independent redox activity, which needs further investigation (33, 59).

## Other biochemical processes associated with ferroptosis

In addition to iron, lipid peroxidation, and GPX4, other biochemical pathways are also essential for ferroptosis. One of them is the pentose phosphate pathway, which produces nicotinamide adenine dinucleotide phosphate (NADPH). NADPH is necessary for the catalytic activity of glutathione reductase, an enzyme that catalyses the conversion of GSSG to GSH. Another pathway is transsulphuration, which allows cysteine synthesis *de novo* in case of deficient extracellular cystine uptake by the glutamate/cysteine antiporter Xc^-^system (60). Lipid metabolism and the mevalonate pathway are also associated with the ferroptosis. In addition to ferroptosis inducers, ferroptosis inhibitors deserve particular attention. They can be classified as iron chelators (e.g. deferoxamine, cyclipirox, deferiprone), lipophilic antioxidants (vitamin E, butylated hydroxytoluene, XJB-5-131, liproxstatin-1, ferrostatin-1), LOX inhibitors (baicalein, zileuton), and deuterated polyunsaturated fatty acids, all of which prevent lipid peroxidation. Literature also mentions glutaminolysis inhibitors, protein synthesis inhibitor cycloheximide (which reduces beta-mercaptoethanol), and neurotransmitter dopamine (20). Some of these inducers and inhibitors of ferroptosis are also suitable for use *in vivo* (e.g. sorafenib and iron chelators).

### Possible physiological and pathological roles of ferroptosis

Little is known about the role of ferroptosis in the development of tissues and organs. Studies have shown that L-ROS levels are increased in embryonic tissues undergoing cell death and that the process can be controlled with GPX4 and lipophilic antioxidants (19, 61). This suggests that ferroptosis is important for maintaining tissue integrity and general homeostasis, although its precise developmental role is still unclear.

Impaired ferroptosis is associated with various pathological conditions such as malignancies, neurodegenerative diseases, ischaemia/reperfusion, acute kidney disease, liver and heart diseases, haemochromatosis, (62, 63), and neuropsychiatric illnesses (such as bipolar disorders, depression, schizophrenia, and Huntington’s disease) (64, 65). Many of these conditions are also accompanied by iron, glutamate, or GSH imbalance and increased lipid peroxidation (66).

*In vitro* and animal studies investigating the role of ferroptosis in tumourigenesis indicate that cancer cell lines derived from the brain, ovary, kidney, bone tissue, and soft tissue are susceptible to ferroptosis, whereas the lines derived from the pancreas, breast, stomach, and upper respiratory system are not (30, 67). These differences in sensitivity to ferroptosis have been attributed to differences in the basal metabolic state of these particular cell types, especially in lipid metabolism. Ferroptosis in cancer cells seems to be promoted by tumour suppressor gene *p53*, NADPH-oxidase (NOX), HMOX-1 and inhibited by miR-137 and transcriptional factors Nrf2 and *p53* (10, 68).

Either way, they directly or indirectly target iron metabolism or lipid peroxidation and show a potential for genetic or pharmacological interventions utilising ferroptosis to eliminate malignant cells and treat different types of cancer. However, it has yet to be established in which cancer types ferroptotic therapy would be effective.

Malignant cells usually have high concentrations of iron and are consequently in persistent oxidative stress (69). This is why some cancers contain somatic mutations in the Nrf2/Keap 1 pathway to enhance transcription of antioxidant enzymes (19, 70, 71). As a consequence, ferroptosis is not frequent in the development of cancers. To stimulate ferroptosis as a therapeutic strategy against cancer, the most common therapeutic targets are the system Xc^-^, GPX4, iron-related genes, GSH, coenzyme Q10 (CoQ10), and LOXs (67). Tumour suppressor gene *p53* (more specifically, acetylation-defective mutant *p53^3KR^*) is also known to induce ferroptosis (19) by suppressing the antiporter system Xc^-^component SLC7A11. However, the roles of wild and mutant *p53* in ferroptosis appear to be extremely complex. Depending on the context, different cells and different test conditions can stimulate or suppress ferroptosis by different mechanisms (72, 73, 74, 75). These studies are ongoing.

### Therapeutic role of ferroptosis

The mechanisms controlling ferroptosis have also been studied as therapeutic targets in various pathological conditions. For ferroptosis-based cancer therapy, iron-based nanomaterials have been designed and synthesised in recent times, such as ferumoxytol (otherwise approved by the United States Food and Drug Administration for iron deficiency therapy) and amorphous iron nanoparticles. Due to certain disadvantages of iron-based nanomaterials, other metals with multiple oxidation states (e.g. manganese dioxide-coated mesoporous silica nanoparticles) and GPX4-inhibiting nanomaterials (e.g. metal-organic network coated on the surface of polyethylenimine/*p53* plasmid complex) are also being tested (74).

Chemotherapeutics (e.g. doxorubicin/adriamycin, cytarabine/ara-C, cisplatin) in combination with erastin show a significantly synergistic antitumor activity (75). Other drugs with the ability to stimulate ferroptosis, such as sulfasalazine, cisplatin, artesunate, sorafenib, lapatinib, salinomycin, and ironomycin are still being tested on cell lines of various cancers (10).

The role of ferroptosis in the treatment of cancer, however, is not yet sufficiently clear. According to Krysko et al. (76), tumour cell death by ferroptosis is a “double-edged sword”. Namely, ferroptotic cells can activate antitumour immune response through immunogenic cell death and thus enhance the effects of anticancer therapy. However, they can also suppress antitumor immune response and contribute to tumour progression. At this stage, no definitive conclusions can be drawn as to whether ferroptosis is an immunogenic or immunosuppressive form of cell death. This is why some authors call for further basic research on animal models (77).

### Need for ferroptosis biomarkers

To confirm the findings of ferroptosis studies, those *in vivo* in particular, it is necessary to define reliable molecular biomarkers of this process. Studies conducted so far are mainly based on the use of different inducers and on monitoring the effects of ferroptosis inhibitors and increased L-ROS values (19). A reliable and specific biomarker has not yet been defined. Candidates include increased expression of prostaglandin E synthase mRNA 2 (PTGS2), glutathione-specific gamma-glutamylcyclotransferase 1, and HMOX 1, which have been reported following erastin-induced ferroptosis (30, 78). Other potential ferroptosis biomarkers could be higher activity of cyclooxygenase 2 (30, 31), higher MDA, and lower NADPH levels (79, 80, 81). Various methodological approaches have been used *in vitro* to determine cell viability, most commonly flow cytometry (30, 82), followed by the measurement of intracellular iron using a specific dye and GSH depletion (83).

## Conclusion

The main feature of ferroptosis is the accumulation of L-ROS in the cell membrane and organelles through iron-dependent lipid peroxidation and inadequate activity of GPX4. There are indications that this form of cell death is relevant in a variety of physiological and pathophysiological contexts and that it could be used to target some cancers.

However, current knowledge is still insufficient and does not answer what role ferroptosis has in the normal development of the organism, how ferroptosis signalling pathways are controlled, what role does iron play, what actually happens to cell membranes after L-ROS accumulation, is LOX essential for the process, does ferroptosis include other enzymes to which iron is a cofactor, and what role the secondary products of lipid peroxidation play.

So far, the most useful information regarding ferroptosis originates from *in vitro* studies that elucidate additional molecular mechanisms and signalling pathways involved in ferroptosis. Further studies are needed to clarify the remaining uncertainties and help transfer new knowledge to clinical settings.

Furthermore, to understand ferroptosis in *in vivo* conditions we need more specific and reliable biomarkers under normal or pathological conditions. This would help us to predict the susceptibility or resistance of certain diseases to ferroptosis and learn how to modulate this process to establish effective therapeutic strategies.

## Abbreviations

AA: arachidonic acid
ACD: accidental cell death
ACSL4: acyl-CoA long chain synthetase 4
AdA: adrenic acid
AIFM1: apoptosis inducing factor mitochondria-1
ATP: adenosine triphosphate
CASP: caspase
CIL56: caspase independent lethal 56
CoQ10: coenzyme Q10
DAMPs: damage-associated molecular patterns
DMT1: divalent metal transporter 1
DNA: deoxyribonucleic acid
FIN56: ferroptosis inducer 56
GPX4: glutathione peroxidase-4
GR: glutathione reductase
GSH: reduced glutathione
GSSG: oxidised glutathione
HMGB1: high-mobility group box 1
HMOX1: haem-oxygenase-1
4HNE: 4-hydroxynonenal
H_2_O_2–_: hydrogen peroxide
IPP: isopentenyl pyrophosphate
IRP-IRE: iron responsive protein-iron responsive element
L-O*: alkoxyl radical
L-OO*: peroxyl radical
L-OOHs: lipid-hydroperoxides
LOX: lipoxygenase
L-ROS: lipid reactive oxygen species
LIP: labile iron pool
LPCAT3: lysophosphatidylcholine acyltransferase 3
MAPK: mitogen-activated protein kinase
MAP1LC3B: microtubule-associated protein 1 light chain 3 beta
MDA: malondialdehyde
miR-137: microRNA-137
NAD: nicotinamide adenine dinucleotide
NADP: nicotinamide adenine dinucleotide phosphate
NADPH: reduced nicotinamide adenine dinucleotide phosphate
NCCD: Nomenclature Committee on Cell Death
NCOA4: autophagic nuclear receptor coactivator 4
NETs: neutrophil extracellular traps
NOX-NADPH: oxidase; nicotine amide adenine dinucleotide phosphate-oxidase
NTBI: non-transferrin-bound iron
*O_2-_: superoxide radical
*OH: hydroxyl radical
p53: tumour suppressor gene p53
PARP1: poly(ADP-ribose) polymerase 1
PCD: programmed cell death
PCBPs: poly-rC binding chaperone protein
PHGPx: phospholipid hydroperoxide glutathione peroxidase
PE: phosphatidylethanolamine
PLs: phospholipids
PUFAs: polyunsaturated fatty acids
PUFA(PE)-OH: lipid alcohol
PL-PUFA(PE)-OOHs: polyunsaturated fatty acids containing phospholipid hydroperoxides
PUFA-PLs: phospholipid acetylated with polyunsaturated fatty acids
PTGS2: prostaglandin E synthase mRNA 2
RSL3: RAS-synthetic lethal 3
RCD: regulated cell death
RNA: ribonucleic acid
RIPK1: receptor interacting serine/threonine protein kinase 1
SLC7A11: cysteine/glutamate antiporter xCT
Se: selenocysteine
Tf: transferrin
TfR1: transferrin receptor-1
TFAM: mitochondrial transcription factor A
